# Dietary phosphorus source reprograms the kidney-intestine axis to dictate calcium-phosphorus efficiency and egg production in aging hens

**DOI:** 10.1186/s40104-026-01506-8

**Published:** 2026-10-12

**Authors:** Astrid Lissette Barreto Sánchez, Minyahel Tilahun, Guiping Zhao, Haijun Zhang

**Affiliations:** 1 Key Laboratory of Feed Biotechnology, Ministry of Agriculture and Rural Affairs, Institute of Feed Research, Chinese Academy of Agricultural Sciences, Beijing, 100081 China; 2 College of Agriculture and Natural Resources, Wolkite University, 07, Wolkite, Ethiopia; 3 State Key Laboratory of Animal Nutrition, Institute of Animal Sciences, Chinese Academy of Agricultural Sciences, Beijing, 100193 China

**Keywords:** Dicalcium phosphate, Gene expression, Layer hens, Metabolic efficiency, Mineral homeostasis, Tricalcium phosphate

## Abstract

**Background:**

The decline in egg production in aging hens poses a significant challenge to sustainable poultry production. Given that evidence suggests the phosphorus source affects late‑lay performance, we tested the hypothesis that the dietary phosphorus source is a decisive regulator sustaining performance by reprogramming systemic mineral homeostasis. Using a systems biology approach, we analyzed the coordinated expression of eleven key mineral transporter genes across the duodenum, jejunum, ileum, and kidney of hens fed dicalcium phosphate (DCP) or tricalcium phosphate (TCP).

**Results:**

During weeks 5–8, hens fed DCP showed significant declines in egg production (β = −3.11/week, *P* < 0.001) and egg weight (β = −0.47 g/week, *P* = 0.049), unlike the stable TCP group. TCP induced an efficient "precision conservation" phenotype, characterized by the co-upregulation of key duodenal phosphorus and calcium transporters (*NaPi-IIb*,* PiT2; NCX1*,* PMCA1b*), a gene set identified as the top predictor of performance. Conversely, DCP triggered a costly "compensatory salvage" state, scattering expression to duodenal calcium channels (*TRPV5*, *TRPV6*) and renal compensatory genes (*NCX1*,* CALB1*). Network analysis pinpointed these renal genes as a central, performance-limiting hub.

**Conclusion:**

We conclude that TCP sustains production by programming a systemically efficient homeostatic strategy, thereby explaining the established performance benefit at a molecular level. This establishes a new principle for layer nutrition: phosphorus source selection must consider its role as a physiological signal that modulates homeostasis. This strategy mitigates age-related decline and enhances resource allocation to production.

**Supplementary Information:**

The online version contains supplementary material available at https://doi.org/10.1186/s40104-026-01506-8.

## Introduction

The global egg industry, a cornerstone of sustainable animal protein production, faces a significant challenge in the persistent decline in egg production and shell quality in aging laying hens [1, 2]. A key factor underpinning this decline is the progressive dysregulation of calcium and phosphorus homeostasis, as the hen's physiological systems struggle to meet the immense, daily mineral demand for eggshell formation [3]. While calcium metabolism has been extensively studied, the role of dietary phosphorus sources, a critical and costly component of laying hen diets, in modulating the underlying molecular pathways during this critical late-lay period remains poorly understood [4, 5]. A deeper investigation into how different phosphorus sources influence the very machinery of mineral absorption and transport is therefore essential for developing targeted nutritional strategies to support the aging hen.

Mineral homeostasis is orchestrated by a complex network of intestinal and renal transporters [6, 7]. Key players include the transient receptor potential vanilloid channels *TRPV5* and *TRPV6* for intestinal calcium uptake [8–10], the sodium-phosphate cotransporters *NaPi-IIb* (intestine) and *NaPi-IIa* (kidney) for phosphorus [11, 12], and the plasma membrane calcium ATPase *PMCA1b* for calcium export [13, 14]. In aging, this system is further challenged by changes in the vitamin D endocrine system, including altered expression of the vitamin D receptor (*VDR*) and the catabolic enzyme *CYP24A1* [15], which can disrupt the delicate balance between calcium and phosphorus availability [16, 17]. However, how dietary phosphorus source orchestrates multi-tissue gene expression and metabolic efficiency during aging remains unknown.

Dicalcium phosphate (DCP) and tricalcium phosphate (TCP) are widely used supplements in poultry diets [18, 19]. These sources differ not only in their phosphorus content but also in their calcium-to-phosphorus ratio and potentially their solubility and bioavailability in the digestive tract [20–22]. These physicochemical differences may be perceived differently at a cellular level, thereby triggering distinct gene expression programs that either promote efficient mineral utilization or induce metabolically costly compensatory mechanisms [23, 24]. However, it remains unknown whether these properties trigger isolated gene expression changes or a fundamental, system-wide reprogramming of mineral homeostasis pathways in critical tissues like the intestine and kidney.

The aging hen is a model of heightened metabolic demand. The well-documented age-related decline in production and feed utilization performance is paralleled by changes in the expression of renal and intestinal transporters, suggesting a system under increasing strain where resources are diverted from production to costly homeostatic maintenance [25, 26]. A dietary strategy that can alleviate this strain by optimizing the gene expression profile for mineral transport could have profound implications for sustaining late-lay production output [5, 27]. The translational significance of these molecular pathways is validated by production performance metrics, where efficient phosphorus utilization correlates with sustained egg output and feed efficiency [28]. Furthermore, the stable performance of TCP-fed hens compared to the declines observed in DCP-fed hens confirms that molecular efficiency translates to production resilience [29]. This establishes a direct link between dietary phosphorus source, systemic gene regulation, and practical laying performance. We propose that the source of dietary phosphorus is not merely a supplier of a nutrient but a potent signal that can rewire metabolic pathways. Ultimately, it determines the allocation of resources and the efficiency of production.

Recent evidence confirms that TCP supports superior egg production and bone strength in aging hens compared to DCP [19]. However, the molecular mechanisms underlying the differences in production performance and feed utilization efficiency remain unknown. To address this mechanistic gap, we employed a systems biology approach, moving beyond single-gene analysis to examine the coordinated expression of eleven key genes across the primary tissues of mineral handling: the duodenum, jejunum, ileum, and kidney. By integrating this gene expression data with detailed performance metrics and employing advanced statistical models like Random Forest regression, we sought to test our central hypothesis and move from correlation to causation, identifying the most influential genes and tissues driving the observed performances.

We hypothesized that an optimized dietary phosphorus source sustains egg production in aging hens by orchestrating a systemically efficient state of mineral homeostasis, thereby lowering metabolic load and redirecting resources toward reproduction. This study investigates three central hypotheses: (1) an optimized dietary phosphorus source enhances the coordinated expression of key mineral transporter genes across intestinal segments and the kidney, establishing a more efficient systemic regulatory state; (2) this multi-tissue gene expression signature serves as the primary, causal driver of production outcomes, identifiable through advanced statistical modeling; and (3) the consequent reduction in metabolic load from homeostatic compensation empirically translates to superior late‑lay performance. Thus, we propose that the phosphorus source acts as a signal that rewires mineral homeostasis to sustain reproductive output.

## Materials and methods

### Ethics approval

The protocol for this study was reviewed and approved by the Institutional Animal Care and Use Committee at the Institute of Feed Research of the Chinese Academy of Agricultural Sciences (IFR-CAAS No. 20220507).

### Experimental animals and dietary design

A total of 576 Hy-Line Brown laying hens at 45 weeks of age were used. The experiment was conducted at the Zhuozhou Test Base, Feed Research Institute, Chinese Academy of Agricultural Sciences (Zhuozhou, China), where the hens had been reared before the trail. The hens were randomly assigned to 8 treatment groups in a completely randomized design, with 6 replicates of 12 hens per treatment. The experiment followed a 2 × 4 factorial arrangement evaluating two phosphorus sources, DCP and TCP, across four non-phytate phosphorus (NPP) supplementation levels (0.07%, 0.14%, 0.21%, and 0.28%). The present study focuses on comparing the two phosphorus sources (DCP vs. TCP) pooled across these levels; detailed effects of individual NPP levels have been reported separately [19]. Basal diets were formulated to meet or exceed nutrient requirements specified by China's poultry feeding standards (NY/T33–2004; ICS 65.020.30; B43) [30], with detailed composition provided in Supplementary Table S1. The analyzed NPP levels of the test diets ranged from 0.17% to 0.38%, covering a spectrum from deficient to adequate relative to the 0.32% NPP recommended by the standard. This range was selected to evaluate the efficacy of DCP and TCP under physiologically relevant phosphorus conditions. The relative bioavailability of TCP was 205.45% of DCP (Supplementary Fig. S1). The analyzed nutrient values (Table S1) were used in all statistical analyses. Small deviations between calculated and analyzed NPP values (≤ 0.01%) are within the expected range of analytical variation, as defined by AAFCO (Association of American Feed Control Officials) guidelines for phosphorus analysis [31]. The study included a 2-week adaptation period followed by a 12-week experimental period.

### Data collection and performance parameters

Production performance data were collected daily and summarized weekly throughout the 12-week experimental period. Key parameters included:

- Egg production (EP, %) calculated as (total eggs produced/number of hens) × 100
- Average egg weight (AEW, g) measured using a digital balance
- Average daily feed intake (ADFI, g/hen/d)
- Feed conversion ratio (FCR, g feed/g egg)

Linear regression was used to evaluate weekly trends in production parameters. The regression coefficient (β) represents the average weekly change in each parameter over the specified period, with negative values indicating a decline and positive values indicating an increase.

### Tissue sampling and RNA extraction

At the conclusion of the experimental period, one hen per replicate (*n* = 6 per treatment) was selected for tissue collection. Kidney, duodenum, jejunum, and ileum tissues were immediately collected, snap-frozen in liquid nitrogen, and stored at −80 °C until analysis [32]. Total RNA was extracted from the kidney, duodenum, jejunum, and ileum using TRIzol^®^ Reagent (Invitrogen, Carlsbad, CA, USA) according to the manufacturer's instructions with a homogenization step. Briefly, 25 mg of each tissue sample was homogenized in 1 mL of TRIzol using a multi-sample tissue grinder (Tissuelyser-48, Jingxin^®^, Shanghai, China) at 70 Hz/s for 1 min. Following homogenization, chloroform was added to the mixture, which was then vigorously mixed and centrifuged to achieve phase separation. The aqueous phase containing the RNA was carefully transferred to a new tube. RNA was precipitated by the addition of isopropanol, and the resulting pellet was washed with 75% ethanol, air-dried, and finally resuspended in RNase-free water. RNA concentration and purity were determined using a NanoDrop2000c spectrophotometer (Thermo Scientific, Wilmington, USA), with all samples exhibiting A_260_/A_280_ ratios between 1.8 and 2.0. RNA integrity was confirmed by agarose gel electrophoresis [33], and stored at −80 °C for future analyses. All procedures were conducted on ice to preserve RNA integrity, following established protocols [34].

### Quantitative PCR analysis

We analyzed eleven key genes involved in calcium and phosphorus homeostasis: *TRPV6*, *TRPV5*, *CALB1*, *NCX1*, *PMCA1b*, *NaPi-IIa*, *NaPi-IIb*, *PiT1*, *PiT2*, *VDR*, and *CYP24A1*. Complementary DNA was synthesized from total RNA samples using the Transcript All-in-One First-Strand cDNA Synthesis SuperMix for qPCR (One-Step gDNA Removal) System (TransGen, Beijing, China) in accordance with the manufacturer's protocols. Quantitative PCR was performed using an ABI 7500 system (Applied Biosystems, Foster City, CA, USA) with FastReal qPCR Premix (SYBR Green) (TianGen^®^, Beijing, China). Each 10 µL reaction mixture contained 1.5 µL of cDNA, 0.3 µL of forward primer, 0.3 µL of reverse primer, 5 µL of SYBR Green dye, 0.2 µL of ROX reference dye, and 2.7 µL of RNase-free water. Reaction conditions consisted of initial denaturation at 95 °C for 2 min, followed by 40 cycles of 95 °C for 10 s and 60 °C for 25 s. Gene expression levels were normalized to the reference gene β-actin and calculated using the 2^−ΔΔCt^ method [35]. The reference gene β-actin was selected based on its validated stability in avian intestinal and renal tissues [36]. Primer sequences for the housekeeping and target genes are provided in Supplementary Table S2.

### Statistical analysis

All statistical analyses were conducted in R version 4.5.1 [37]. The analytical framework included: Data were checked for normality using Shapiro–Wilk tests and for homogeneity of variances using Levene's tests [38]. Outliers were identified using Grubbs' test and removed when appropriate. Data transformation (log or square root) was applied when necessary to meet model assumptions.

For gene expression data, we employed linear mixed-effects models using the *lme4* package [39] with phosphorus source and phosphorus level as fixed effects, and replicate as a random effect. Tissue-specific analyses were conducted using two-way ANOVA followed by Tukey's HSD post-hoc tests for pairwise comparisons when significant main effects were detected. False discovery rate (FDR) correction was applied using the Benjamini–Hochberg procedure [40] to control for multiple comparisons.

Spearman correlation analysis was performed using the *Hmisc* package [41] to assess relationships between gene expression patterns across tissues and performance parameters. Correlation matrices were visualized using the *corrplot* package [42], with significance levels set at FDR-adjusted *P* < 0.05.

To determine the relative contribution of each gene to laying performance, we employed Random Forest regression models using the *randomForest* package [43]. Separate models were built for each performance trait (EP, AEW, ADFI, FCR) using normalized gene expression data from all four tissues as predictors. Model performance was evaluated using out-of-bag (OOB) error estimation. Variable importance was calculated based on the percentage increase in mean squared error (%IncMSE) when each predictor was permuted, with higher %IncMSE values indicating greater importance for predicting the outcome variable. Statistical significance was defined as FDR-adjusted *P* < 0.05 for all analyses, with trends discussed at *P* < 0.10. Data are presented as mean ± standard error of the mean (SEM) unless otherwise specified.

## Results

### Production performance and feed utilization

**Fig. 1:**
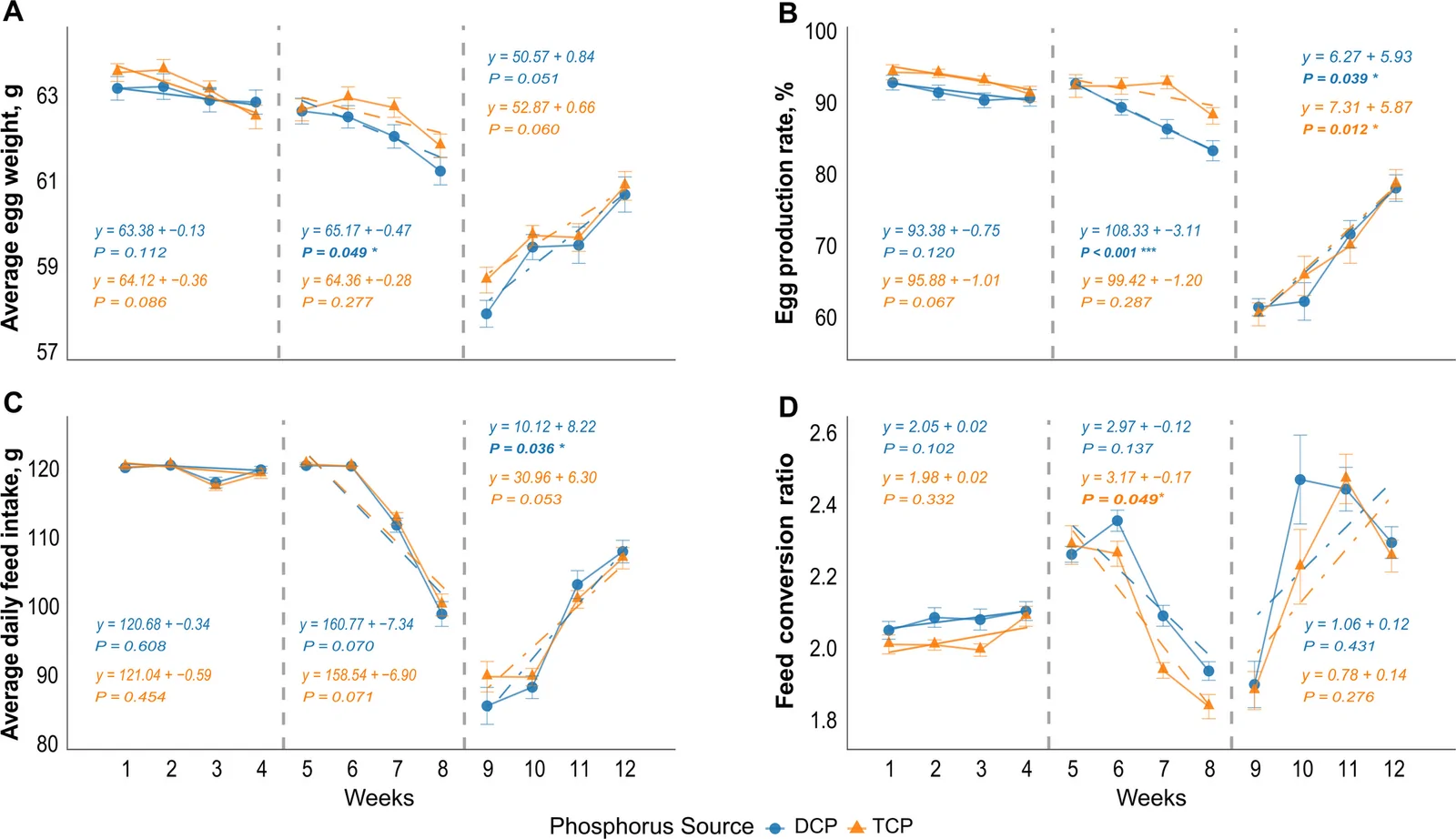
Production performance of aging laying hens fed dicalcium phosphate (DCP) or tricalcium phosphate (TCP) over a 12-week trial. **A** Weekly average egg weight (AEW). **B** Weekly egg production (EP). **C** Weekly average daily feed intake (ADFI). **D** Weekly feed conversion ratio (FCR). Fitted regression lines are shown for each treatment (DCP: blue dashed line; TCP: orange dashed line). The grey background panels denote the three trial phases (weeks 1–4, 5–8, and 9–12). Significance levels: ^*^P < 0.05, ^**^P < 0.01, ^***^P < 0.001

Average egg weight was stable in the first four weeks for both treatments, but diverged between weeks 5 and 8. Hens fed DCP exhibited a significant decline in AEW (β = −0.47 g/week, *P* = 0.049), while the TCP group showed no significant trend (*P* = 0.277) (Fig. 1A). A borderline-significant increase was observed in both groups from weeks 9 to 12. Egg production showed the most critical treatment effect. During weeks 5–8, the DCP group suffered a sharp, highly significant decline (β = −3.11 eggs/week, *P* < 0.001), whereas production in the TCP group remained stable with no significant decrease (*P* = 0.287) (Fig. 1B). Both groups showed a significant production increase from weeks 9 to 12 from a lower baseline. Average daily feed intake remained generally stable during weeks 1 to 8, despite a transient, non-significant decline from weeks 5 to 8, but increased significantly in the DCP group during weeks 9 to 12 (β = + 8.22 g/week, *P* = 0.036) (Fig. 1C). The TCP group showed a similar but borderline significant increasing trend (*P* = 0.053). Feed conversion ratio showed a significant improving trend (decreasing FCR; β = −0.17, *P* = 0.049) during weeks 5 to 8 for the TCP group (Fig. 1D). No other significant weekly trends in FCR were detected for either treatment.

### Phosphorus source reprograms mineral transporters

**Fig. 2:**
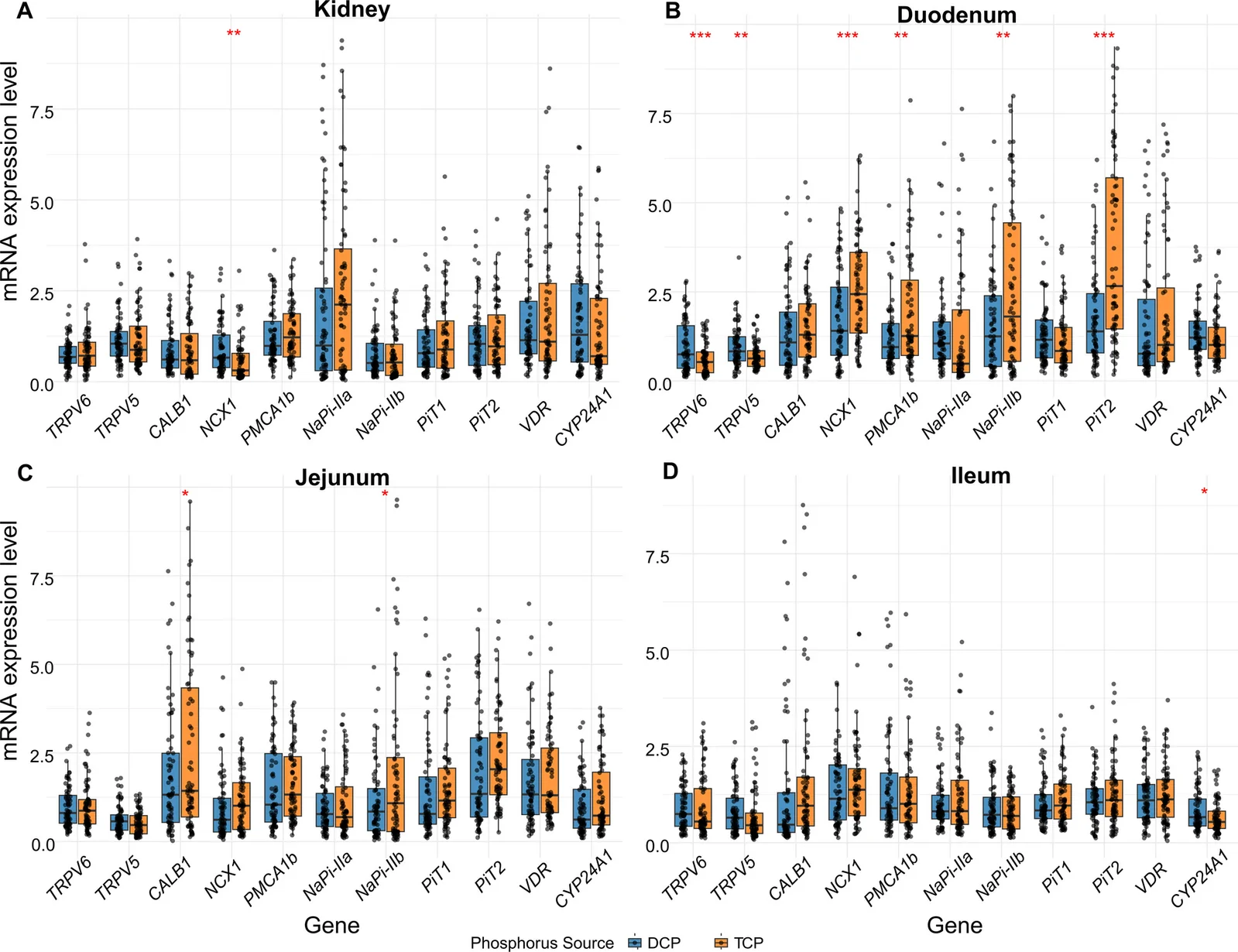
Tissue-specific gene expression comparing phosphorus sources. Expression levels of calcium and phosphorus-related genes (*TRPV6, TRPV5, CALB1, NCX1, PMCA1b, NaPi-IIa, NaPi-IIb, PiT1, PiT2, VDR, CYP24A1*) in (**A**) Kidney, (**B**) Duodenum, (**C**) Jejunum, and (**D**) Ileum of laying hens fed diets supplemented with either dicalcium phosphate (DCP, blue) or tricalcium phosphate (TCP, orange). Data are presented as boxplots with individual data points. Statistical significance (Student's *t*-test) between phosphorus sources for each gene is indicated above the plots (^*^P < 0.05, ^**^P < 0.01, ^***^P < 0.001)

Gene expression analysis of eleven calcium and phosphorus-related genes revealed distinct regulatory patterns in the kidney, duodenum, jejunum, and ileum driven by dietary phosphorus source (Fig. 2). The most pronounced effects occurred in the duodenum. The TCP diet elicited a strong duodenal response, significantly upregulating *NCX1* (*P* < 0.001), *PMCA1b* (*P* < 0.01), *NaPi-IIb* (*P* < 0.01), and *PiT2* (*P* < 0.001). In the jejunum, *CALB1* and *NaPi-IIb* were also significantly upregulated (*P* < 0.05). Furthermore, TCP feeding was associated with consistent, though non-significant, increases in *VDR* expression across all four tissues, alongside modest elevations in renal *TRPV5, PMCA1b, PiT2,* and *NaPi-IIa*, indicating a broader gene expression profile consistent with enhanced mineral transport capacity. Conversely, the DCP diet significantly increased the expression of duodenal calcium channels *TRPV6* (*P* < 0.001) and *TRPV5* (*P* < 0.01), renal *NCX1* (*P* < 0.01), and ileal *CYP24A1* (*P* < 0.05) (Fig. 2A–D).

### Differential gene co-expression networks in DCP and TCP groups

**Fig. 3:**
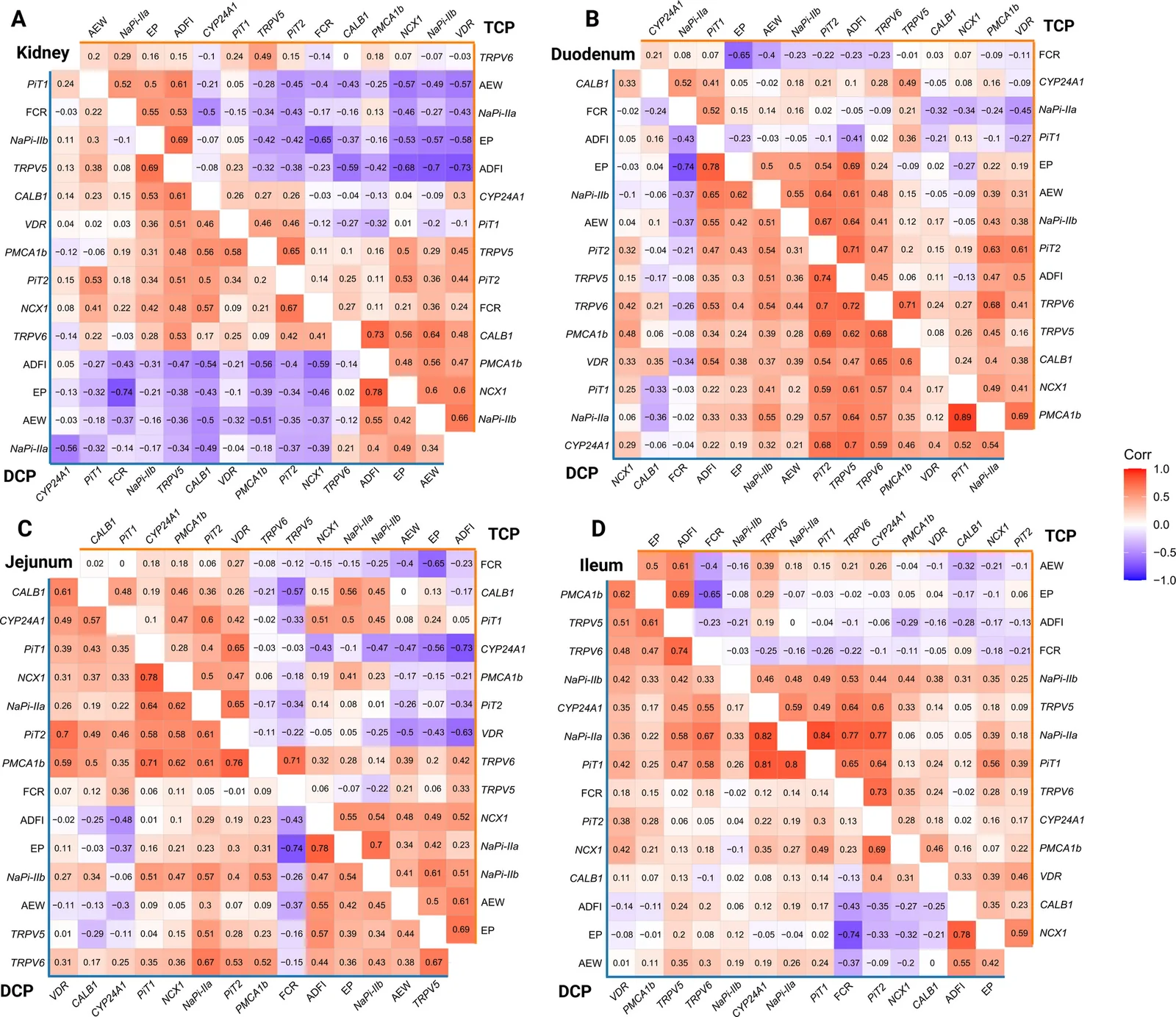
Gene expression correlation of dicalcium phosphate (DCP) and tricalcium phosphate (TCP) groups across four tissues. The heat maps display pairwise Spearman correlation coefficients between gene expression levels in the kidney (**A**), duodenum (**B**), jejunum (**C**), and ileum (**D**) tissues from the two groups. Correlation values are represented with a color gradient, where shades of red indicate positive correlations (near +1) and shades of blue indicate negative correlations (near −1). The correlation plot bounded by the blue line represents DCP, and the orange line represents TCP. Significant correlations are identified based on FDR-adjusted *P* < 0.05

Gene expression correlation analysis across four tissues revealed distinct patterns between the DCP and TCP groups (Fig. 3). In the kidney, DCP correlated positively between key transporters such as *TRPV5/NaPi-IIb* and EP/ADFI, while showing negative correlations for EP/FCR and *NaPi-IIa/CYP24A1*. Conversely, TCP exhibited positive correlations like *CALB1/PMCA1b* and *TRPV5/PiT2*, alongside broad negative associations between performance metrics (AEW, EP, ADFI) and most calcium and phosphorus transporters (Fig. 3A). This tissue-specific divergence continued in the duodenum, where DCP showed a strong positive *NaPi-IIa/PiT1* correlation, whereas TCP demonstrated positive links for *PMCA1b* with *VDR* and *TRPV6*, alongside multiple negative correlations involving FCR (Fig. 3B).

Within the jejunum and ileum, further group-specific relationships emerged. Jejunal DCP presented positive correlations among phosphate transporters and vitamin D pathway components (*PiT1/PMCA1b, PiT2/VDR*), while TCP correlated *VDR* with *CYP24A1* and *PiT2*, and *TRPV5* with *TRPV6* (Fig. 3C). In the ileum, DCP showed strong positive associations, particularly within the *CYP24A1-PiT1-NaPi-IIa* network and between *TRPV5/TRPV6*. For TCP in this segment, *NaPi-IIa* correlated strongly with *PiT1, TRPV6*, and *CYP24A1*. Notably, a consistent negative correlation between EP and FCR was observed across all tissues and in both groups, while a positive EP/ADFI correlation was similarly maintained throughout.

### Systemic organization of mineral homeostasis

**Fig. 4:**
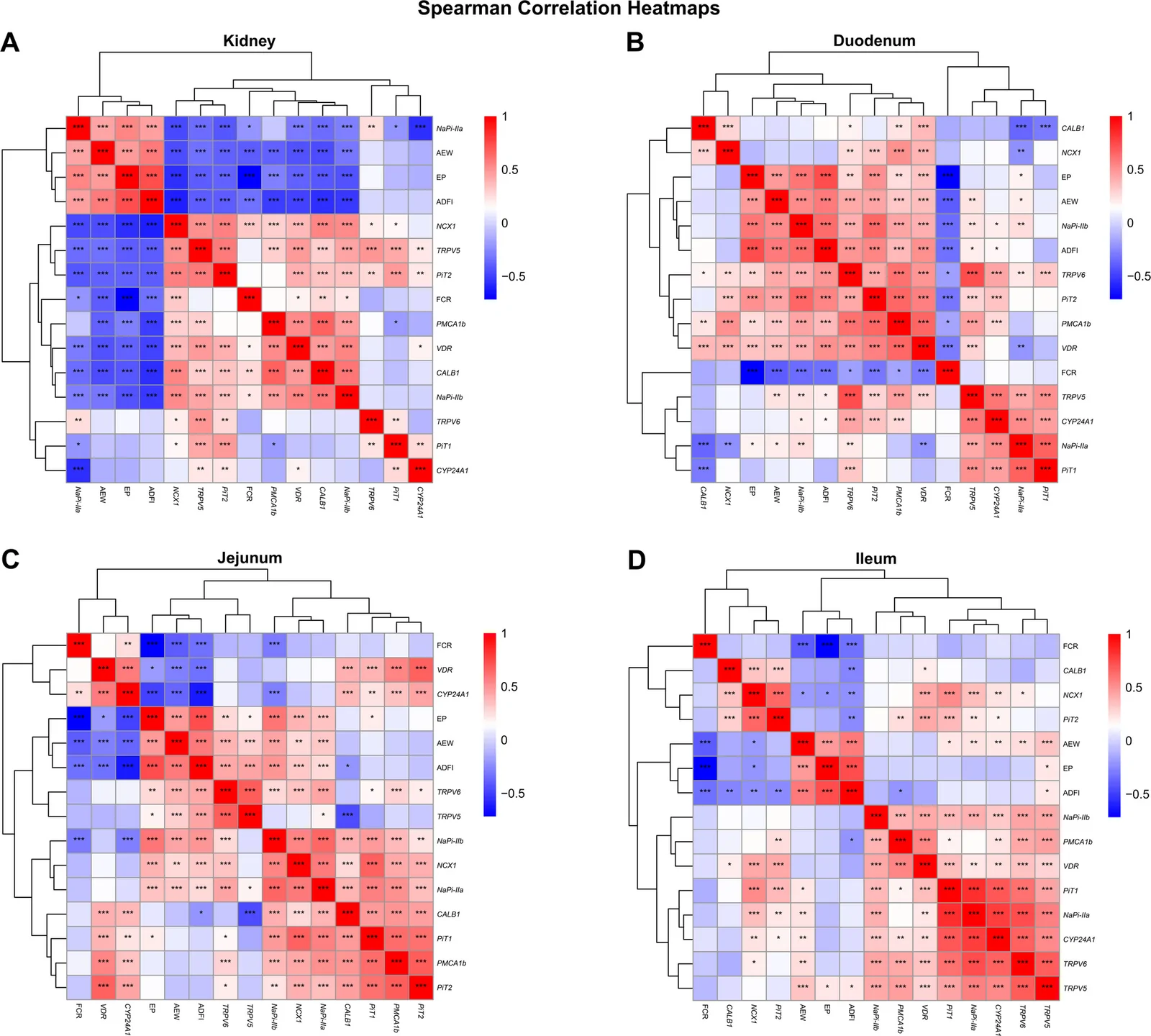
Systemic mineral homeostasis network revealed by hierarchical clustering of gene co-expression patterns across tissues. **A–D** The matrix displays pairwise Spearman correlation coefficients between four tissue parts for genes involved in mineral homeostasis. Coefficients were considered significant at FDR-adjusted *P* < 0.05. Rows and columns were reordered by hierarchical clustering (dendrograms on top and left) based on correlation-derived distance, grouping tissues with similar co-expression profiles. Positive (blue) and negative (red) correlations indicate coordinated or antagonistic gene regulation across the system

To further elucidate the systemic organization of mineral homeostasis, a hierarchical co-expression analysis was performed to visualize associations and clustering (Modules are presented separately for each cluster across the tissues (Fig. 4) and the total (Fig. S2)) between the eleven genes and production parameters across all tissues.

In the kidney, the network resolved into three distinct modules (Fig. 4A). Module 1 linked production traits (ADFI, EP, AEW) with the phosphate reabsorber *NaPi-IIa* in a positive correlation cluster. Module 2 exhibited a strong internal negative correlation structure among the transporters *NCX1, TRPV5, *and* PiT2*. Module 3 grouped the feed efficiency metric FCR positively with the genes *PMCA1b, VDR, CALB1, *and* NaPi-IIb*.

The duodenal network formed three modules (Fig. 4B). Module 1 showed a strong positive correlation between production metrics (EP, AEW, and ADFI) and the phosphate transporter *NaPi-IIb*. Module 2 was defined by a coordinated positive association among the calcium transporter genes *TRPV6 *and* PMCA1b*, the phosphate transporter *PiT2*, and the regulatory gene *VDR*. Module 3 contained two positive correlation pairs: *TRPV5* with *CYP24A1*, and *NaPi-IIa* with *PiT1*.

The jejunum and ileum each formed two primary modules (Fig. 4C and D). In the jejunum, Module 1 contained the production traits (EP, AEW, ADFI) in a cluster of negative internal correlations. Module 2 revealed coordinated expression among key transporters, showing strong positive correlations between *NaPi-IIa* and *NCX1*, and among *CALB1, PiT1, PMCA1b*, and *PiT2*. In the ileum, Module 1 grouped production traits (AEW, EP, ADFI) in a strong positive cluster. Module 2 contained distinct positive regulatory units: one linking *PMCA1b* and *VDR* with *NaPi-IIb*, another pairing *NaPi-IIa *and* CYP24A1* with *PiT1*, and a third correlating *TRPV6* with *TRPV5*.

Hierarchical clustering of pooled data across all tissues revealed ten systemic co-association modules (Fig. S2). Key patterns included: 1) an antagonistic renal module (Module 2) where transporters *NCX1, CALB1*, and *NaPi-IIb* were negatively correlated with duodenal phosphate transporters (*PiT2, NaPi-IIb*) and ADFI, and inversely linked to FCR; 2) cohesive intestinal networks (Modules 6 and 7) combining jejunal phosphate (*PiT1, PiT2, NaPi-IIa*) and calcium (*PMCA1b, NCX1*) transporters with ileal calcium channels (*TRPV5*, *TRPV6*); and 3) a performance-negative module (Module 8) featuring a negative correlation between duodenal *PiT2* and production metrics (AEW, ADFI, EP). Other modules showed renal *CYP24A1* negatively associated with performance (Module 3), and ileal *CYP24A1* positively correlated with *NaPi-IIa* and *PiT1* (Module 10).

### Divergent systemic integration of diets

**Fig. 5:**
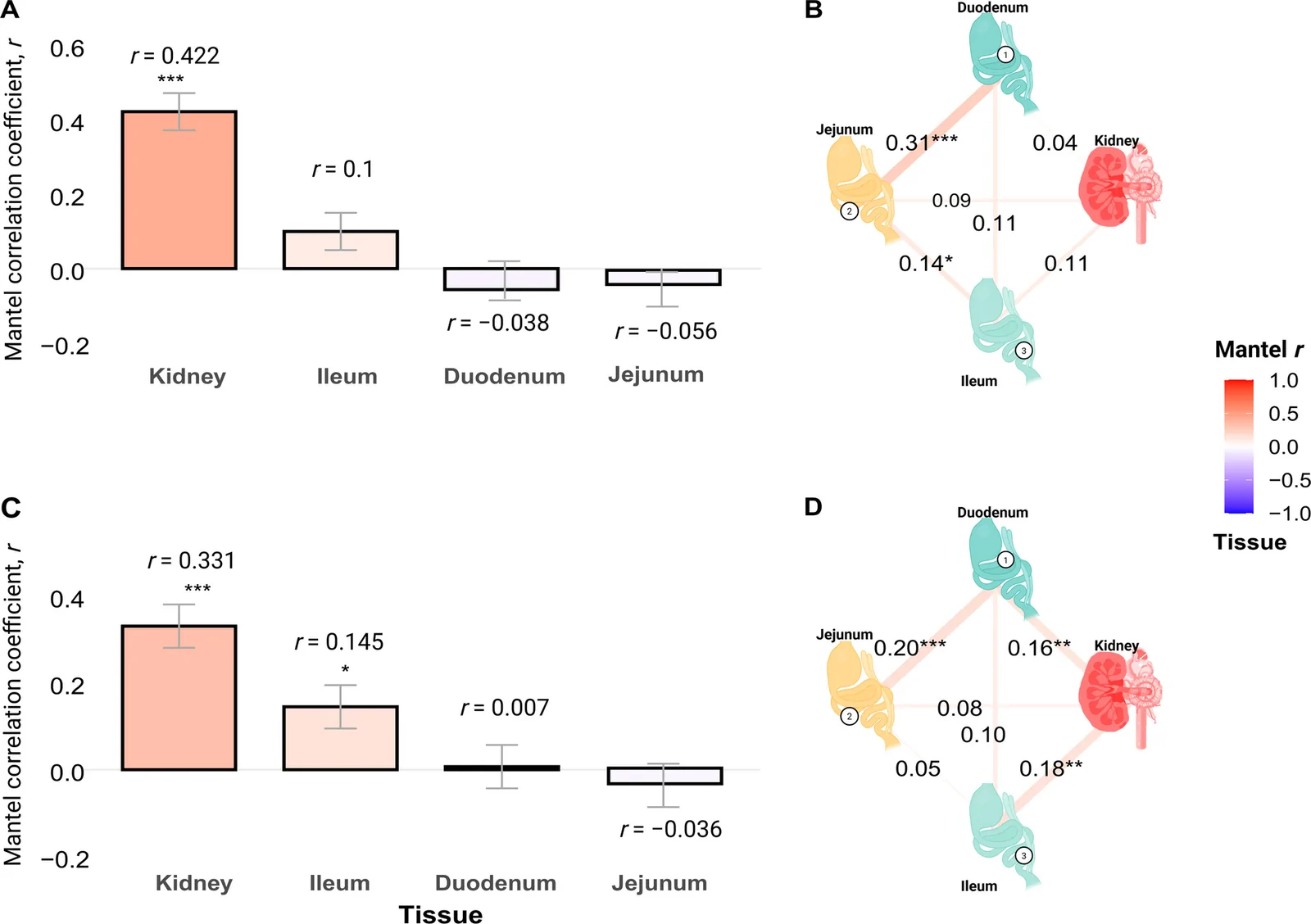
Mantel test analysis of systemic network integration. Pairwise correlations between tissue-specific gene expression networks and their associations with production metrics for DCP-fed (**A** and **B**) and TCP-fed (**C** and **D**) hens. Panels A and C show tissue-performance correlations; panels B and D show tissue-tissue correlations. Line color indicates direction (red: positive; blue: negative), thickness represents strength, and asterisks denote significance (^*^P < 0.05, ^**^P < 0.01, ^***^P < 0.001)

In the DCP group, a low but statistically significant positive correlation was found between the kidney gene network and performance parameters (*r* = 0.42, *P* < 0.001). The ileum showed a low, non-significant positive correlation, while the duodenum and jejunum networks exhibited negative, non-significant correlations with performance (Fig. 5A). The TCP group also showed a significant positive correlation between the kidney network and performance (*r* = 0.33, *P* < 0.001), and the ileum exhibited a significant but negligible positive correlation (*r* = 0.15, *P* < 0.05). The jejunum network again showed a negative, non-significant correlation (Fig. 5C). In a pooled analysis across both diets, the kidney network maintained a significant positive correlation with performance (*r* = 0.35, *P* < 0.001), while the ileum correlation was significant but negligible (*r* = 0.1, *P* < 0.05). The duodenum and jejunum networks showed negative, non-significant correlations (Fig. S3A).

Pairwise analysis revealed distinct patterns of inter-organ connectivity. In the DCP group, a strong, highly significant positive correlation was observed between the duodenum and jejunum (*r* = 0.4, *P* < 0.001), indicating tightly coordinated gene regulation. A significant but weaker correlation was found between the jejunum and ileum (*r* = 0.2, *P* < 0.05), suggesting more moderate functional coupling (Fig. 5B). In contrast, the TCP group exhibited a more extensive network of strong positive correlations. Significant coordination was observed not only between the duodenum and jejunum (*r* = 0.4, *P* < 0.001) but also between the kidney and duodenum, and between the kidney and ileum (*r* = 0.4, *P* < 0.01 for both) (Fig. 5D). This indicates a system-wide integration of gene regulation across intestinal and renal tissues in the TCP group. In the pooled analysis, the duodenum and jejunum remained strongly correlated (*r* = 0.4, *P* < 0.001), and a weaker correlation was present between the kidney and ileum (*r* = 0.2, *P* < 0.05). Other tissue pairs were not significantly correlated (Fig. S3B).

### A dietary switch in homeostatic strategy

**Fig. 6:**
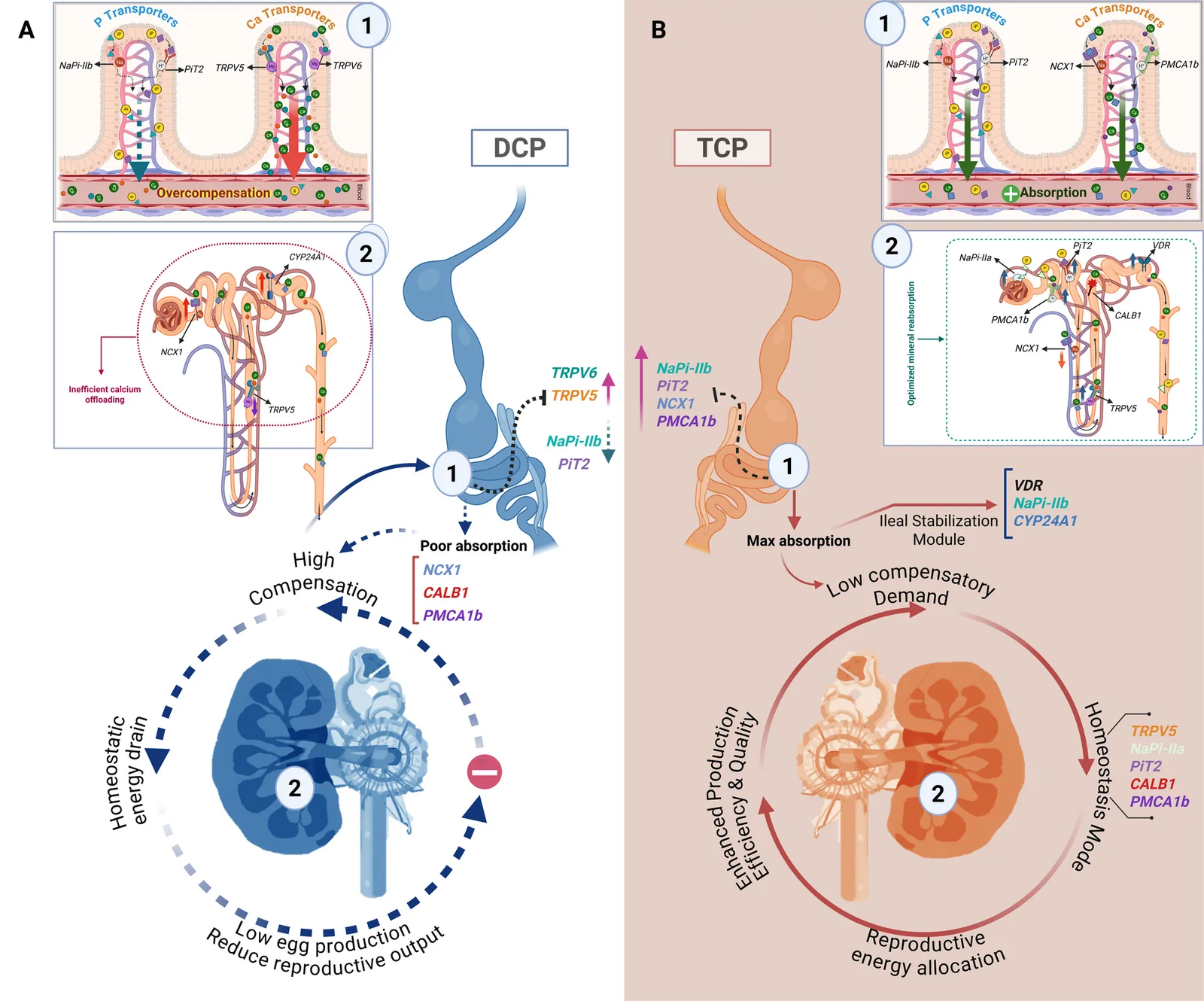
A mechanistic model for phosphorus-source dependent regulation of calcium homeostasis in the aging hen. **A** The "compensatory salvage" state (DCP diet). A signal of intestinal mineral insufficiency disrupts systemic coordination, downregulating the renal precision pathway and upregulating a high-capacity salvage program. This costly, reactive state reflects systemic calcium stress and diverts metabolic resources from production. **B** The "precision conservation" state (TCP diet). Efficient intestinal absorption establishes systemic sufficiency, enabling the kidney to employ a high-affinity, vitamin D-dependent reabsorption pathway. This proactive, low-cost state promotes coordinated homeostasis and directs metabolic resources toward sustained egg production. DCP and TCP represent dicalcium phosphate and tricalcium phosphate, respectively. Created with BioRender.com

Integrated gene expression and network analyses reveal a distinct, feed-dependent physiological mechanism that explains the performance disparity between dietary groups (Fig. 6). This mechanism pivots on the fundamental contrast between a state of systemically coordinated sufficiency and one of discordant, compensatory salvage.

In hens fed DCP, an imbalanced duodenal response, marked by a predominant upregulation of calcium channels (*TRPV5*, *TRPV6*) without proportional induction of phosphate importers (Fig. 2B), signals a perceived Ca:P imbalance. This inefficient primary absorption forces a systemic shift, provoking a high compensatory demand on post-absorptive organs. The kidney responds by activating a "compensatory salvage" pathway, significantly upregulating the high-capacity exporter *NCX1*, while ileal *CYP24A1* expression increases, potentially modulating vitamin D signaling (Fig. 2A and D). This adaptation, reflected in isolated duodenum-jejunum connectivity and a weak kidney-performance linkage (Fig. 5B), represents a less selective, energetically costly effort to reclaim minerals. The consequent homeostatic energy drain diverts metabolic resources, directly leading to lower egg production and reduced reproductive output.

Conversely, in hens fed TCP, a pronounced duodenal program characterized by coordinated upregulation of phosphate importers (*NaPi-IIb, PiT2*) and calcium exporters (*NCX1, PMCA1b*) establishes a foundation for efficient, balanced mineral absorption (Fig. 2B). This robust intestinal priming minimizes the post-absorptive compensatory load. This state enables the kidney to operate a "precision conservation" pathway, supported by modest, coordinated elevations in key transporters (*TRPV5, PMCA1b, PiT2, NaPi-IIa*) and *VDR*. Crucially, the kidney’s correlation profile within the TCP group shows a distinct pattern: egg production is positively associated with the phosphate reabsorber *NaPi-IIa* but negatively associated with other salvage transporters (*TRPV5, PMCA1b, PiT2*), indicating enhanced productive efficiency (Fig. 3A). This state is defined by system-wide integration, evidenced by strong positive gene correlations linking the kidney network to intestinal segments and performance outcomes (Fig. 5D). The concerted, tissue-coordinated action facilitates selective mineral handling, promoting a virtuous cycle of stable homeostasis with minimal metabolic cost.

Thus, TCP sustains production by promoting a coherent, low-cost homeostatic state across organs, whereas DCP triggers a discordant, high-cost compensatory mode that diverts energy from production, providing a clear mechanistic explanation for the observed differences in late-lay hen performance.

### Duodenum as the primary predictive node for performance

**Fig. 7:**
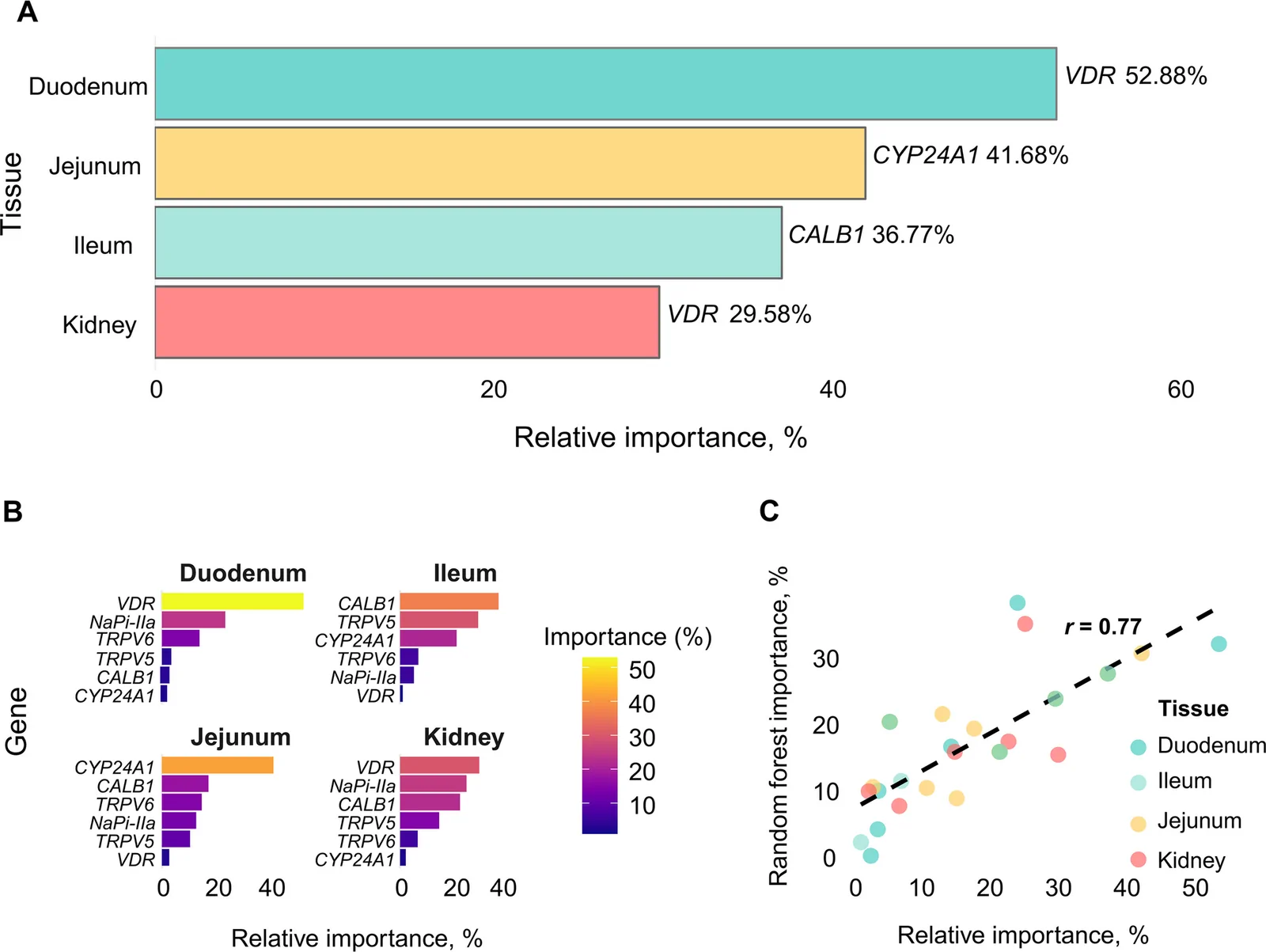
Tissue gene contributions to egg production. **A** Top-performing gene from each tissue. **B** Comprehensive tissue gene importance profiles. **C** Validation of importance rankings across statistical methods

To identify the key tissue-specific genetic regulators of production performance, we performed Random Forest regression analysis (Fig. 7). This revealed distinct primary drivers: duodenal *VDR* was the strongest overall predictor (52.88% relative importance), followed by jejunal *CYP24A1* (41.68%), ileal *CALB1* (36.77%), and renal *VDR* (29.58%) (Fig. 7A). The overall gene importance profiles across the tested tissues demonstrate the correlation of each gene with egg production efficiency (Fig. 7B). A scatter plot illustrates the correlation between the random forest feature importance rankings and the relative importance profiles for each tissue (Fig. 7C). The robustness of these predictive rankings was confirmed by a strong positive correlation (*r* = 0.77) with an independent validation method, underscoring the reliability of these key genes as major determinants linking organ-specific mineral homeostasis to overall hen productivity.

**Fig. 8:**
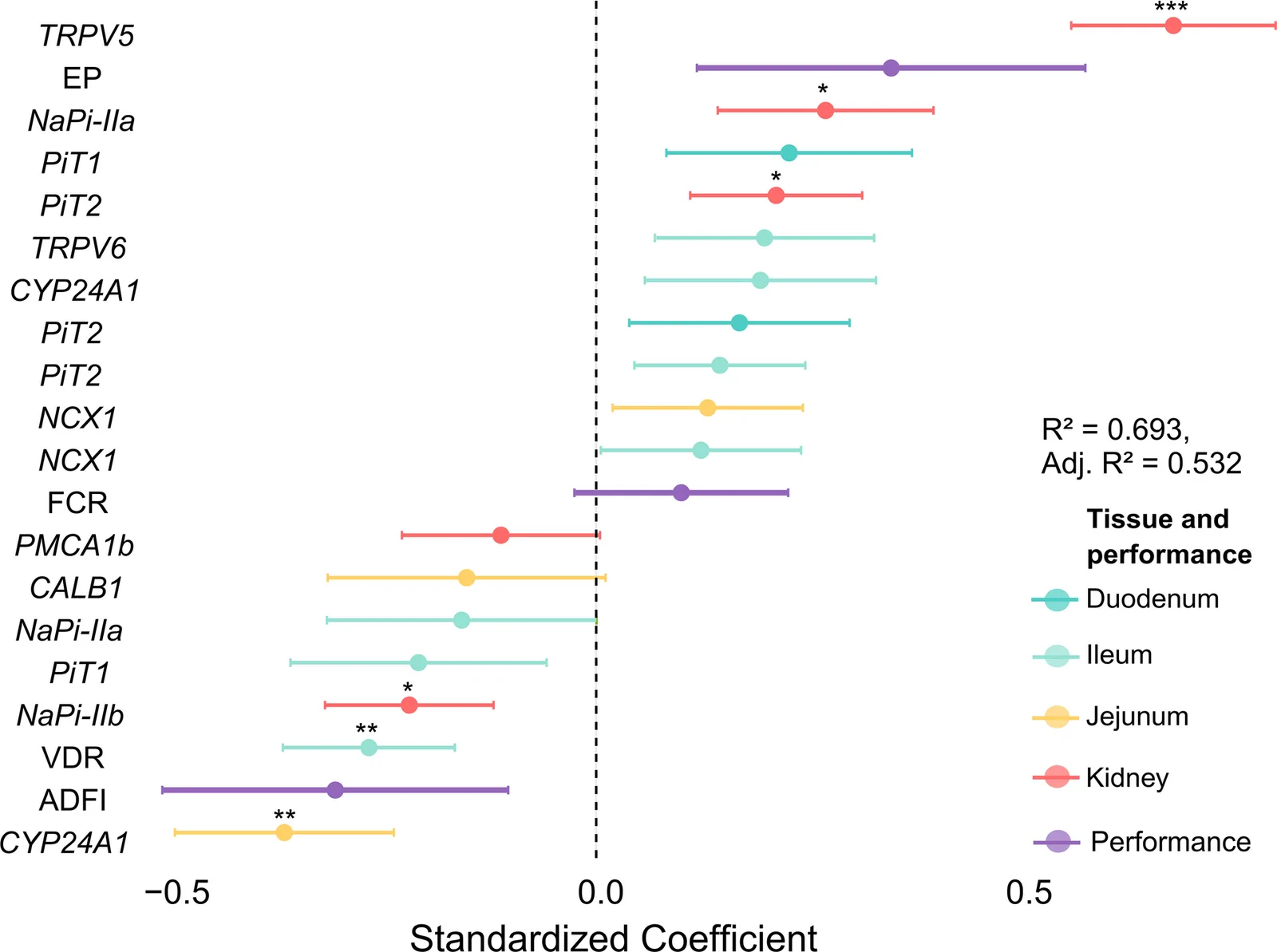
Forest plot of tissue-specific gene effects on performance outcomes. Performance metrics (EP, FCR, ADFI) are displayed on the *y*-axis, with their standardized coefficient on the *x*-axis. Effect sizes (points) and 95% confidence intervals (horizontal lines) show the direction and magnitude of associations. Points to the right of the vertical line (zero) indicate positive associations with performance. Statistical significance levels are indicated by ^*^P < 0.05, ^**^P < 0.01, ^***^P < 0.001

The analysis of standardized coefficients among various tissues sheds light on their relative impacts on gene expression (Fig. 8). The kidney exhibits several coefficients, notably for *TRPV5* and *NaPi-IIa*, which are statistically significant (*P* < 0.001, *P* < 0.01; respectively). In comparison, the duodenum, jejunum, and ileum show a mix of coefficients, but none reach the same level of significance as those associated with the kidney.

## Discussion

This study provides a comprehensive molecular analysis of how DCP and TCP differentially reprogram the systemic gene networks governing calcium and phosphorus homeostasis in aging laying hens. Our results demonstrate that these sources do not merely alter nutrient supply but initiate fundamentally distinct physiological states. The TCP diet orchestrates a "precision conservation" state, characterized by coordinated, efficient gene expression in the intestine that enables high-fidelity renal reabsorption, a program predictively linked to superior laying performance. In contrast, the DCP diet triggers a "compensatory salvage" state, marked by maladaptive intestinal shifts and a high-cost renal adaptation, reflecting systemic mineral strain. Our findings establish that phosphorus source is not just a dietary component but a critical signaling input that determines metabolic priority, acting through three integrated pillars: optimizing duodenal transport efficiency, maintaining systemic mineral homeostasis via kidney precision, and ultimately redirecting metabolic resources from costly compensation toward sustained egg production. This molecular understanding provides a robust scientific foundation for precision nutrition strategies aimed at enhancing the longevity and productivity of the aging commercial flock.

### The duodenum as a decoder of phosphorus source

Our findings provide direct genetic evidence that the dietary phosphorus source is decoded by the duodenum, initiating distinct gene expression programs that dictate systemic mineral efficiency. We demonstrate that TCP upregulates a coordinated duodenal "phosphorus efficiency module" (*NaPi-IIb, PiT2, NCX1, PMCA1b*), a program strongly correlated with superior egg production (Fig. 3B). In contrast, DCP elicits a discordant response, predominantly stimulating calcium salvage channels (*TRPV5*, *TRPV6*) without proportional phosphate transporter induction (Fig. 2B). This source-specific reprogramming directly addresses a critical unresolved mechanism, providing the missing link between phosphorus source bioavailability [22] and the duodenum's role as the principal site for vitamin D-regulated absorption [44, 45]. Consequently, the duodenum functions not merely as a passive absorptive site, but as the primary processor of a dietary signal that sets the efficiency of the entire mineral homeostasis network.

This TCP-induced signature reflects a program optimized for efficient, stoichiometrically balanced co-transport, aligning with the proximal intestine's dominant absorptive role [46, 47], and extending prior knowledge by directly linking a specific gene profile to bioavailability and performance outcomes, a connection not explicitly established in earlier work [48]. Conversely, the imbalanced DCP response suggests a perceived insufficiency in bioavailable phosphate, a condition known to dysregulate normal absorption coupling [16]. This molecular signal of intestinal insufficiency compels a fundamental shift in whole-body mineral homeostasis, specifically reprogramming the functional kidney-intestine axis [49, 50].

The efficiency established by TCP-primed intestinal state has cascading systemic benefits. The TCP-induced program creates a streamlined pathway that minimizes intracellular imbalance and energetic costs (Fig. 5D). While prior research indicates efficient intestinal absorption reduces renal workload [51], our study provides clear gene expression evidence of this trade-off in poultry. Critically, this molecular efficiency is directly reflected in the birds' physiology. Hens on the TCP diet did not experience the significant mid-trial decline in egg production or egg weight seen in the DCP group (Fig. 1A and B), supporting the model that their homeostatic system was operating without costly strain. The efficient TCP program aligns with superior feed conversion and sustained egg mass [5, 20]. Conversely, the inefficient DCP signature likely forces compensatory absorption [23], which manifested physiologically as the sharp, significant decline in egg production during weeks 5–8 (Fig. 1B), a clear sign of systemic metabolic stress correlating with physiological strain (Fig. 5B). Therefore, the duodenal gene profile dictates whether the system operates in a state of efficient coordination or costly compensation, providing a mechanistic explanation for the link between phosphorus source and productivity [3, 27]. The contrast with studies showing minimal effects [52, 53] may be attributed to differences in hen age or basal diet, highlighting the importance of the physiological context examined here.

### Reprogramming the kidney-intestine axis

Our findings establish that dietary phosphorus source acts as a decisive signal, systemically reprogramming the kidney-intestine axis to determine whole-organism metabolic efficiency. This reprogramming is directly evidenced by the contrasting TCP and DCP phenotypes (Fig. 6). The TCP diet initiated a state of coordinated sufficiency. The efficient intestinal priming by a balanced duodenal transport module [11, 14] was permissively linked to a renal "precision conservation" pathway (*TRPV5, PMCA1b*). Crucially, the Mantel test reveals strong positive correlations integrating the kidney gene network with both the duodenum and ileum (Fig. 5D). This statistical validation of cross-organ communication defines a tightly coupled, integrated axis that minimizes the metabolic cost of homeostasis. Consequently, TCP fosters a virtuous cycle of efficient mineral handling, which directs energy toward sustained production and serves as a crucial strategy for mitigating age-related dysregulation [3, 26].

In contrast, the DCP diet initiates a physiologically costly state by disrupting this systemic coordination. This is evidenced by a distinct molecular phenotype, characterized by a “compensatory salvage” program (Fig. 6). In response to a perceived Ca:P imbalance signaled by the intestine, the kidney engaged a high-capacity salvage pathway, marked by upregulation of the exporter *NCX1* and the vitamin D catabolic enzyme *CYP24A1,* classic adaptations to correct a perceived mineral deficit [17, 54]. Network analysis statistically validates the systemic disconnection inherent to this state, revealing strong gene correlations confined to an intestinal loop, with the kidney functionally isolated (Fig. 5B). This renal shift initiates a high-energy salvage effort, diverting metabolic resources from production and providing the direct molecular explanation for the concurrent declines in egg production and egg weight observed in DCP-fed hens during the critical mid-trial period (Fig. 1A and B), thus establishing a direct explanation for DCP's inferior performance, aligning with the principle that elevated maintenance costs limit production in aging livestock [55, 56].

The established functions of these genes confirm that the TCP and DCP signatures represent coherent, source-specific physiological programs [57, 58]. The biological mechanism, therefore, is a dietary switch between a proactive, low-cost "precision conservation" strategy and a reactive, high-cost "compensatory salvage" strategy. The statistical proof of systemic integration versus disconnection provides definitive evidence for these contrasting organizational states. Therefore, the ultimate performance outcome in the aging hen is dictated by the metabolic efficiency of the activated homeostatic strategy, not merely phosphorus intake.

### A dietary switch between efficiency and salvage

The results reveal that dietary phosphorus source dictates one of two opposing physiological strategies. TCP initiates a proactive "precision conservation" strategy for coordinated, low-cost efficiency. We term this state “precision conservation” to highlight its coordinated, metabolically efficient mineral handling strategy. This state is directly evidenced by a harmonized molecular phenotype, including a balanced duodenal transport module and a renal system where high egg production positively correlates with the specific phosphate reabsorber *NaPi-IIa*. The co-upregulation of duodenal genes establishes a frontline for balanced mineral co-transport [21], while the renal correlation profile reflects selective, vitamin D-dependent reabsorption [11, 13]. The biological mechanism is a feed-induced state of perceived sufficiency that minimizes metabolic overhead by ensuring efficient primary absorption, thereby minimizing the need for energetically expensive renal salvage [19]. Consequently, TCP sustains late-lay production not merely by supplying phosphorus but by activating a physiologically economical strategy that redirects metabolic resources from costly homeostasis toward reproduction, aligning with the principle of metabolic prioritization [59, 60].

Conversely, the DCP diet triggers a reactive "compensatory salvage" strategy, which we define as a high-cost, reactive program that reflects systemic mineral strain and diverts resources from production. This is directly evidenced by a discordant molecular phenotype: an isolated duodenal upregulation of calcium salvage channels without matched phosphate importers, coupled with a renal program broadly upregulating the high-capacity exporter *NCX1*. This imbalanced gene expression reflects a physiological shift; the induction of *TRPV5* and *TRPV6* is a documented response to hypocalcemic challenge, while renal *NCX1* upregulation signifies a shift toward bulk mineral reclamation [15]. The biological mechanism is a feed-induced signal of mineral insufficiency that fractures the kidney-intestine axis, forcing a high-energy, reactive compensation in the kidney to correct a perceived deficit. The severe energetic drain of this salvage state is directly quantified by the performance data: it coincides precisely with a significant decline in egg production and a spike in feed intake in the DCP group (Fig. 1B and C), as the hen's metabolism is redirected toward homeostasis and away from reproduction. Therefore, the poor performance associated with DCP is mechanistically explained by this energetically expensive salvage mode, which diverts critical metabolic resources from production.

This distinction establishes a core principle: phosphorus source dictates the fundamental efficiency of the mineral regulatory system. The "precision conservation" strategy is a hierarchical, energy-conserving program focused on optimal initial absorption, thereby liberating metabolic resources for production. The "compensatory salvage" strategy is a resource-draining, reactive program that increases systemic workload. Therefore, the superior laying performance with TCP stems from activating a physiologically economical state that redirects energy unequivocally from costly compensation toward sustained reproductive output [5, 25]. This provides a mechanistic explanation, specifically the strategic reprogramming of the kidney-intestine axis, for performance differences that extend beyond simple nutrient bioavailability.

### Toward precision nutrition for the aging hen

The discovery of phosphorus source-specific homeostatic strategies provides a molecular blueprint for transitioning from static nutrient formulation to dynamic physiological programming in aging hens. This is directly evidenced by the superior performance linked to the "precision conservation" gene signature. The improved egg production and feed efficiency observed with TCP in this study are consistent with our previous findings, where we demonstrated that TCP enhances production performance and bone strength in aging hens through differential regulation of bone remodeling genes [19]. The superior bioavailability of TCP (205.45% of DCP; Supplementary Fig. S1) likely contributes to these effects by enhancing phosphorus utilization and supporting metabolic processes essential for egg formation. Specifically, the expression of a coordinated duodenal module (*NaPi-IIb, PiT2, PMCA1b*) shows positive correlation with egg production. Moreover, the stability of egg production and egg mass in TCP-fed hens throughout the trial (Fig. 1A and B) provides the functional validation of this signature's efficacy. Therefore, this duodenal gene signature presents a valuable, sensitive biomarker. In future research, it could be used to screen novel phosphorus sources or dietary additives for their ability to induce a ‘TCP-like’ efficient homeostatic state, moving beyond gross digestibility to assess their ability to induce a systemically efficient state [61, 62].

Conversely, our correlation analysis provides equally critical validation for a novel nutritional target: minimizing renal salvage. The strong negative correlations between key renal genes (*NCX1, CALB1, VDR*) and all performance metrics (Fig. 3A) statistically define compensatory renal work as a measurable drag on productivity. The underlying biological mechanism is that an optimal phosphorus source preemptively reduces the systemic mineral load, thereby preventing the activation of the kidney's costly "compensatory salvage" pathway.

Consequently, the primary goal of phosphorus supplementation should evolve to prioritize sources that promote intestinal efficiency, as defined by this biomarker signature, to support a low-maintenance-load state. These results further reinforce the concept that phosphorus source is not merely a nutrient supply but a physiological signal that modulates systemic mineral homeostasis and reproductive performance in aging hens. This molecular understanding directly addresses the core challenge of aging by identifying key leverage points to sustain production, providing a rational foundation for developing future precision nutrition strategies for the longevity of the layer flock [63, 64]. Beyond phosphorus source optimization, other nutritional interventions, such as supplementation with bioactive peptides, have also been shown to enhance laying performance and egg quality [65], further supporting the broader principle that targeted nutritional strategies can improve productivity in laying hens.

### Limitations and future perspectives

While this study establishes a gene expression framework linking phosphorus source to systemic homeostatic strategy, its correlative nature points to essential avenues for future research. Furthermore, the initial sensory mechanism by which the duodenum discriminates between phosphorus sources remains a key unknown. The applicability of this model under commercial conditions, in different genetic strains, or in the presence of concurrent environmental stresses also warrants investigation.

These limitations, however, define a clear pathway for advancing the field. Future work should integrate targeted proteomics and longitudinal physiological studies to confirm causality and explore interactions with other hallmarks of aging. Ultimately, this work provides the foundational molecular rationale for a paradigm shift in layer nutrition: moving from phosphorus supplementation to the strategic selection of sources that actively program an efficient physiological state, a critical step toward precision nutrition for aging livestock.

## Conclusion

In conclusion, our findings demonstrate that the dietary phosphorus source acts as a decisive physiological signal, reprogramming systemic mineral homeostasis. The optimized source (TCP) promoted a "precision conservation" state, characterized by a coordinated duodenal transporter program (*NaPi-IIb, PiT2, NCX1, PMCA1b*) that reduced renal compensatory load. This enhanced homeostatic efficiency directly translated to superior late‑lay performance, with TCP-fed hens maintaining stable egg production and feed efficiency, while DCP-fed counterparts experienced significant declines. Together, these results confirm that TCP orchestrated a systemically efficient homeostatic state through coordinated multi-tissue gene expression, with the duodenum as a key predictive node and reduced metabolic load sustaining reproductive output. Therefore, selecting a phosphorus source based on its capacity to program efficient mineral networks emerges as a critical strategy to mitigate the age-related performance declines observed in this study and support sustainable egg production.

## Supplementary Information

Additional file 1: Table S1. Basic diet composition and nutritional levels of the study for laying hens (as-fed basis). Table S2. List of primers of target and housekeeping genes for qPCR analysis.

Additional file 2: Fig. S1. Relative bioavailability of TCP compared to DCP. Fig. S2. Correlation matrix of mineral transporter gene expression and production traits pooled across tissues, revealing systemic interactions in mineral homeostasis. Fig. S3. Mantel test pooled analysis of systemic network integration.

## Data Availability

Data will be made available on request.

## Abbreviations

ADFI: Average daily feed intake
AEW: Average egg weight
ANOVA: Analysis of variance
CALB1: Calbindin-D28k (Calcium-binding protein 1)
cDNA: Complementary deoxyribonucleic acid
CYP24A1: Cytochrome P450 family 24 subfamily A member 1
DCP: Dicalcium phosphate
EP: Egg production
FCR: Feed conversion ratio
NaPi-IIa: Sodium-phosphate cotransporter type IIa
NaPi-IIb: Sodium-phosphate cotransporter type IIb
NCX1: Sodium-calcium exchanger 1
NPP: Non-phytate phosphorus
PiT1: Sodium-dependent phosphate transport protein 1
PiT2: Sodium-dependent phosphate transport protein 2
PMCA1b: Plasma membrane calcium-transporting ATPase 1, isoform b
TCP: Tricalcium phosphate
TRPV5: Transient receptor potential vanilloid 5
TRPV6: Transient receptor potential vanilloid 6
VDR: Vitamin D receptor
