# Supplementary material for: Dietary phosphorus source reprograms the kidney-intestine axis to dictate calcium-phosphorus efficiency and egg production in aging hens
Source: J Anim Sci Biotechnol. 2026 Oct 12;17:193. doi: 10.1186/s40104-026-01506-8 (PMC13660273; doi:10.1186/s40104-026-01506-8)
Supplement: Supplementary file 1 [file 40104_2026_1506_MOESM1_ESM.docx]

**Table S1** Basic diet composition and nutritional levels of the study for laying hens (as-fed basis), %

| **Items** | **P supplemented level** | | | | | | | |
| --- | --- | --- | --- | --- | --- | --- | --- | --- |
|  | **Dicalcium phosphate (DCP)** | | | | **Tricalcium phosphate (TCP)** | | | |
|  | **0.07%** | **0.14%** | **0.21%** | **0.28%** | **0.07%** | **0.14%** | **0.21%** | **0.28%** |
| Basal diet |  |  |  |  |  |  |  |  |
| Corn | 63.53 | 63.53 | 63.53 | 63.53 | 63.53 | 63.53 | 63.53 | 63.53 |
| Soybean meal | 24.98 | 24.98 | 24.98 | 24.98 | 24.98 | 24.98 | 24.98 | 24.98 |
| Wheat bran | 0.20 | 0.20 | 0.20 | 0.20 | 0.20 | 0.20 | 0.20 | 0.20 |
| Soybean oil | 0.65 | 0.65 | 0.65 | 0.65 | 0.65 | 0.65 | 0.65 | 0.65 |
| Salt | 0.22 | 0.22 | 0.22 | 0.22 | 0.22 | 0.22 | 0.22 | 0.22 |
| DL-Methionine | 0.10 | 0.10 | 0.10 | 0.10 | 0.10 | 0.10 | 0.10 | 0.10 |
| 50% choline chloride | 0.10 | 0.10 | 0.10 | 0.10 | 0.10 | 0.10 | 0.10 | 0.10 |
| Limestone | 9.23 | 8.89 | 8.73 | 8.49 | 9.12 | 8.78 | 8.43 | 8.08 |
| DCP | 0.39 | 0.79 | 1.18 | 1.57 | - | - | - | - |
| TCP | - | - | - | - | 0.38 | 0.76 | 1.14 | 1.52 |
| Zeolit | 0.23 | 0.33 | 0.19 | 0.04 | 0.60 | 0.56 | 0.53 | 0.50 |
| Premix^1^ | 0.12 | 0.12 | 0.12 | 0.12 | 0.12 | 0.12 | 0.12 | 0.12 |
| Total | 100 | 100 | 100 | 100 | 100 | 100 | 100 | 100 |
| Nutrient levels | | | | | | | | |
| Metabolizable energy, kcal/kg | 2,700 | 2,700 | 2,700 | 2,700 | 2,700 | 2,700 | 2,700 | 2,700 |
| Crude protein (CP)^2^ | 16.50 | 16.50 | 16.50 | 16.50 | 16.50 | 16.50 | 16.50 | 16.50 |
| Calcium^2^ | 3.51 | 3.49 | 3.48 | 3.50 | 3.52 | 3.48 | 3.51 | 3.49 |
| Non-phytate phosphorus level (NPP) ^2^ | 0.17 | 0.24 | 0.33 | 0.38 | 0.18 | 0.25 | 0.34 | 0.38 |
| Total phosphorus (TP)^2^ | 0.48 | 0.54 | 0.62 | 0.67 | 0.48 | 0.55 | 0.62 | 0.67 |
| Sodium (Na) | 0.22 | 0.22 | 0.22 | 0.22 | 0.22 | 0.22 | 0.22 | 0.22 |
| Chloride (Cl) | 0.16 | 0.16 | 0.16 | 0.16 | 0.16 | 0.16 | 0.16 | 0.16 |
| Methionine | 0.37 | 0.37 | 0.37 | 0.37 | 0.37 | 0.37 | 0.37 | 0.37 |
| Lysine | 0.82 | 0.82 | 0.82 | 0.82 | 0.82 | 0.82 | 0.82 | 0.82 |
| Met + Cys | 1.19 | 1.19 | 1.19 | 1.19 | 1.19 | 1.19 | 1.19 | 1.19 |

^1^The premix provided the following per kg of diet: VA 12,500IU, VD3 4,125 IU, VK3 2.0mg, VB1 1.0mg, VB2 8.5mg, VB12 0.02mg, VB6 3.0mg, VE 15 IU, pantothenic acid: 11mg, niacin: 32.5mg, biotin: 0.5mg, folic acid: 1.25mg, Cu: 8mg, Zn: 66mg, Fe: 60mg, Mn: 65mg, Se: 0.3mg, I: 1.0 mg

^2^Analyzed value. Each value is based on determinations made in triplicate

**Table S2** List of primers of target and housekeeping genes for qPCR analysis

| **Gene name** | **GenBank accession ID** | **Primer’s sequence (5' to 3')** |
| --- | --- | --- |
| TRPV5 | XM_004938143.2 | Forward: TGGAACGGACTAAGTCAGAAGTTG  Reverse: CGTTATGGCTGGGATGTTGTT |
| TRPV6 | XM_040655275.2 | Forward: GATCTGTGGGCGAGAGTACG  Reverse: GCCTCGTGACAGTGATGGAA |
| CALB1 | NM_205513.1 | Forward: CAGGGTGTCAAAATGTGTGC  Reverse: GCCAGTTCTGCTCGGTAAAG |
| NCX1 | NM_001079473.1 | Forward: TCACCTTCTTCTTCTTCCCAATCT  Reverse: GCAACCTTTCCGTCCATCTC |
| PMCA1b | XM_015277056 | Forward: TTCAGGTACTCATGTGATGGAAGG  Reverse: CAGCCCCAAGCAAGGTAAAG |
| NaPi-IIa | XM_015293844.1 | Forward: TGGCTAGCTCCTTCCAGATC  Reverse: GAGATGCCGAAGATGAGGGA |
| NaPi-IIb | NM_204474.2 | Forward: GTCCGTTCACTCTGTTGCCT  Reverse: TGGGTCCTCTTCTTGCCTTG |
| PiT1 | XM_015297502.2 | Forward: GCTCGTGGCTTCGTTCTTG  Reverse: GGACCATTTGACGCCTTTCT |
| PiT2 | NM_001305398.1 | Forward: GCAGCAGATACATCAACTC  Reverse: ATTTCCACTCCACCCTC |
| VDR | NM_205098.1 | Forward: CGATGTTCACCTGTCCGTTC  Reverse: CGATGACTTTCTGCTGCTCC |
| CYP24A1 | AF019142.1 | Forward: AAACCCTGGAAAGCCTATCG  Reverse: CCAGTTTCACCACCTCCTTG |
| β-actin | NM_205518 | Forward: ATTGTCCACGCAAATGCTTC  Reverse: AAATAAAGCCATGCCAACTCGTC |

TRPV5, transient receptor potential vanilloid 5; TRPV6, transient receptor potential vanilloid 6; CALB1, calbindin 1; NCX1, sodium‑calcium exchanger 1; PMCA1b, plasma membrane calcium ATPase 1b; NaPi‑IIa, sodium‑phosphate cotransporter type IIa; NaPi‑IIb, sodium‑phosphate cotransporter type IIb; PiT1, sodium‑dependent phosphate transport protein 1; PiT2, sodium‑dependent phosphate transport protein 2; VDR, vitamin D receptor; CYP24A1, cytochrome P450 family 24 subfamily A member 1; β‑actin, beta‑actin
