# Supplementary material for: Dietary phosphorus source reprograms the kidney-intestine axis to dictate calcium-phosphorus efficiency and egg production in aging hens
Source: J Anim Sci Biotechnol. 2026 Oct 12;17:193. doi: 10.1186/s40104-026-01506-8 (PMC13660273; doi:10.1186/s40104-026-01506-8)
Supplement: Supplementary file 2 [file 40104_2026_1506_MOESM2_ESM.docx]

**Fig. S1** Relative bioavailability of TCP compared to DCP. DCP as 100% reference, bars show individual parameter relative bioavailability. Values calculated using slope ratio method from multiple linear regression. All parameters significant at *P* < 0.001

**Fig. S2** **Correlation matrix of mineral transporter gene expression and production traits pooled across tissues, revealing systemic interactions in mineral homeostasis.** The matrix visualizes Spearman correlation coefficients among the expression levels of 11 mineral transporter genes across four tissues (kidney, duodenum, jejunum, ileum) and four production traits (EP, AEW, ADFI, FCR) in aging laying hens. Only significant correlations (FDR‑adjusted *P* < 0.05) are shown, with blue indicating positive and red indicating negative relationships

**Fig. S3** Mantel test pooled analysis of systemic network integration. **A** Correlation between tissue gene networks and performance metrics (AEW, EP, ADFI, FCR). **B** Pairwise correlations between tissue gene expression patterns. Line color indicates direction (red: positive; blue: negative), thickness represents strength, and asterisks show significance (^*^*P* < 0.05, ^**^*P* < 0.01, ^***^*P* < 0.001)
